# Under Pressure: A Case Report of a Diver's Close Encounter With the Bends

**DOI:** 10.7759/cureus.70939

**Published:** 2024-10-06

**Authors:** Shelby Remmel, Neha Chintapally, Juan Enciso

**Affiliations:** 1 Internal Medicine, University of South Florida Morsani College of Medicine, Tampa, USA; 2 Internal Medicine, University of South Florida (USF) Health, Tampa, USA; 3 Cardiology, Medical University of South Carolina, Charleston, USA

**Keywords:** barotrauma, decompression sickness, generalized barotrauma, hyperbaric oxygen, hyperbaric oxygen thetapy, the bends, underwater diving

## Abstract

Generalized barotrauma, also referred to as decompression sickness (DCS), is a condition that occurs when there is a sudden shift in atmospheric pressure. While typically associated with underwater excursions or deep-sea dive encounters, this process can also occur during sudden changes in high altitude or unpressurized air travel. Sudden shifts in atmospheric pressure trigger the formation of nitrogen gas bubbles in the bloodstream that fail to clear from the blood and instead accumulate, leading to an obstruction in circulation. Symptoms of DCS may range from joint and musculoskeletal pain to headaches and even stroke-like symptoms, including visual impairment and altered sensorium. The optimal treatment approach for DCS involves hyperbaric oxygen therapy (HBOT) to allow for the dissolution of nitrogen. However, despite the benefits of HBOT, it is not always readily accessible due to the limited availability of hyperbaric chambers. We present the case of a 50-year-old man diagnosed with acute DCS successfully treated using only high-flow oxygen supplementation.

## Introduction

Decompression sickness (DCS), colloquially referred to as "the bends," is a rare but potentially life-threatening condition that affects countless divers. With an estimated incidence of 0.4 to one case per 10,000 dives, it remains a clinically relevant outcome and concern for diving medicine. DCS occurs when the atmospheric pressure drops below the cumulative partial pressures of oxygen, carbon dioxide, nitrogen, helium, and water vapor in the vascular system [1]. When this happens, gas bubbles can form in the circulation during decompression, leading to mechanical disruption of tissues. The resultant vascular obstruction and ischemia can cause edema and pain, which can mimic symptoms of cerebrovascular accidents [1]. We present the case of a 50-year-old man who exhibited symptoms of underwater barotrauma and was later diagnosed with DCS.

## Case presentation

A 50-year-old military veteran with over 20 years of combined recreational and military service experience and no prior history of DCS arrived at the Emergency Department (ED) with severe pain and noticeable swelling in his right shoulder. He had been engaged in a spearfishing dive, reaching a depth of 70 feet (approximately 21.3 meters). While underwater and handling his spearfishing gun, he experienced a sharp, intense pain in his right shoulder. Despite ascending appropriately, he noticed increasing swelling and worsening pain in the shoulder, which extended to the right anterior chest. The patient had severe limitations in his range of motion without any associated focal numbness or paresthesia. Before this dive and the subsequent retropulsion trauma from the kickback of his weapon, the patient reported a recent history of a mechanical ground-level fall to the right shoulder. Mild musculoskeletal tenderness was reported, but the patient denied any limitations to his strength, range of motion, or activities of daily living.

**Figure 1:**
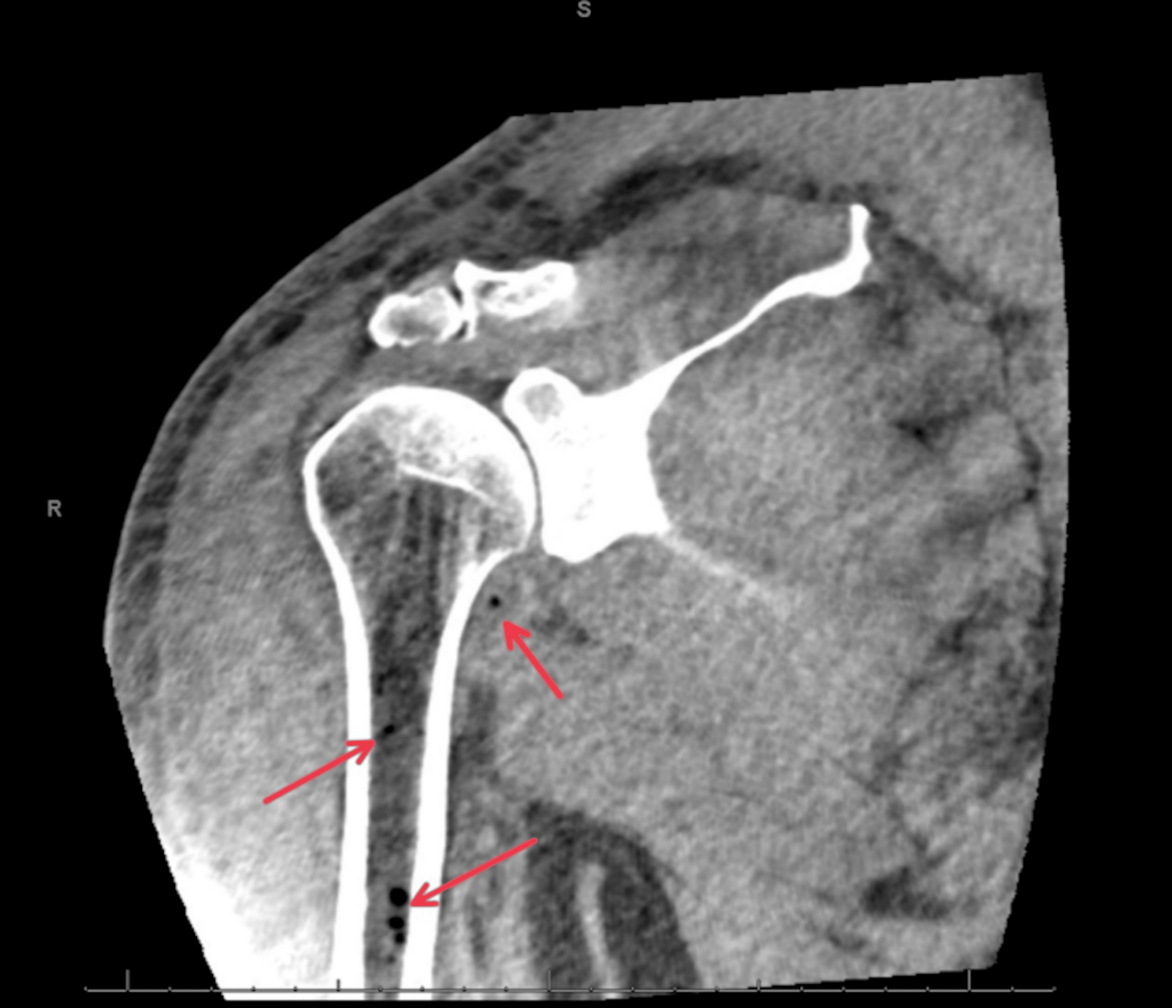
Multiple small hypodense foci in the medullary cavity of the proximal humeral diaphysis.

**Figure 2:**
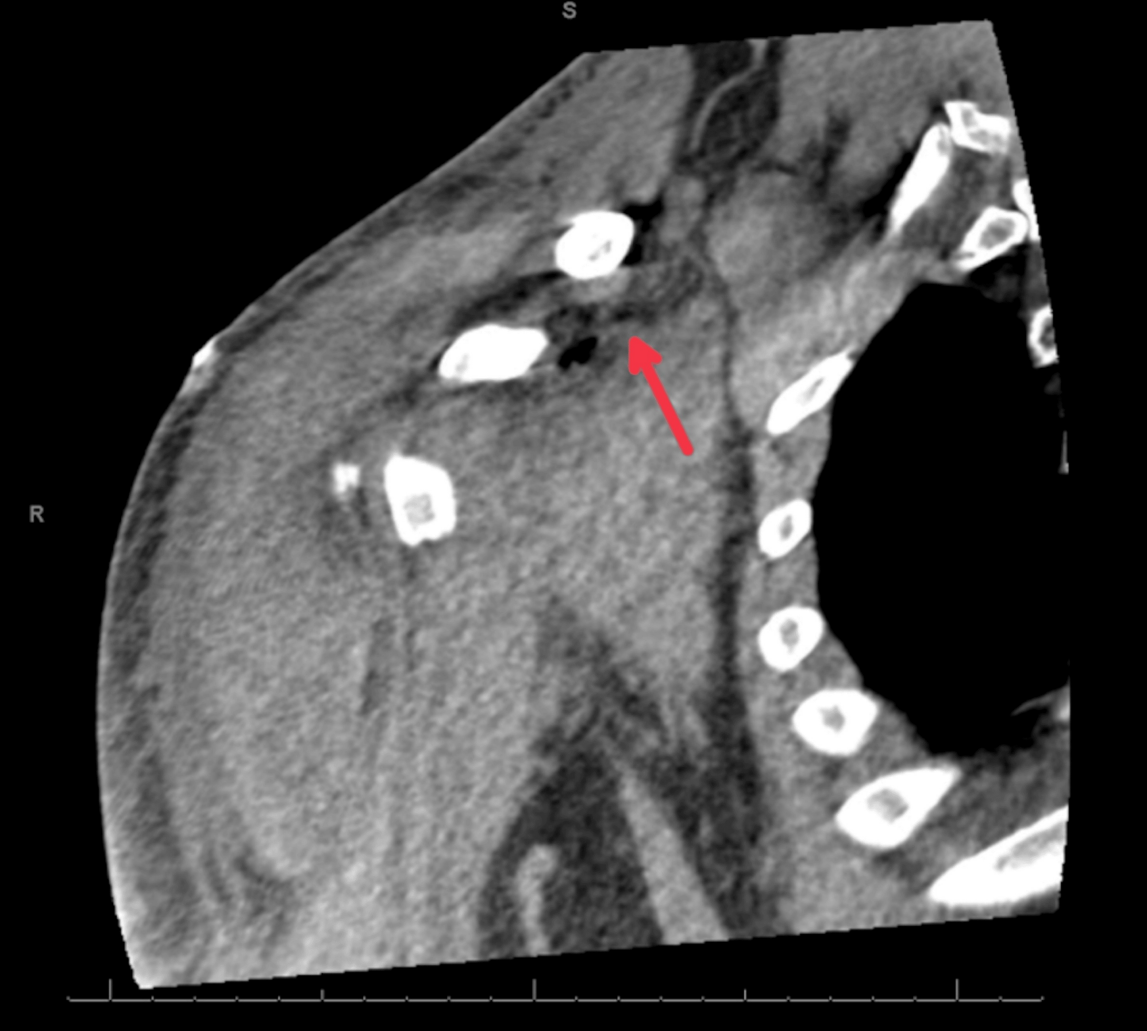
Punctate foci of air in the soft tissues just medial to the base of the coracoid process and punctate focus of air further proximally near the lateral base of the neck.

Following a thorough assessment in the ED, the patient exhibited stable vital signs, and laboratory tests showed acute kidney injury (AKI) related to a previously treated case of nephrolithiasis. An X-ray of the shoulder revealed minor degenerative changes in the acromioclavicular joint. A CT scan simultaneously revealed the presence of gas within the proximal humeral diaphysis (Figure 1) and extensive edema medial to the base of the coracoid process and nearby soft tissues (Figure 2).

Based on the patient's clinical presentation and imaging results, our initial diagnostic considerations included a potentially worrisome indication of DCS, necrotizing fasciitis in the context of underwater barotrauma, or other subcutaneous gas-forming organisms or conditions pertinent to the underwater nature of the injury. To refine our diagnostic approach, we considered our patient's presentation and recent diving history with a meticulous clinical and physical examination to scrutinize potential cutaneous/subcutaneous trauma in conjunction with his clinical presentation and imaging findings. Upon admission, the patient was started on maximum allotted oxygen supplementation via nasal cannula at a rate of 6 L; additionally, due to concerns regarding necrotizing fasciitis, empirical antibiotic therapy was initiated, consisting of vancomycin, piperacillin-tazobactam, and clindamycin (for its antitoxin effects against beta-hemolytic strep). In the absence of an operational hyperbaric oxygen delivery facility in close proximity, the decision was made to manage the patient medically using high-flow oxygen therapy, administering 15 L of 100% O_2_ via a non-rebreather mask as an alternative treatment. While not standard for DCS, this approach has effectively treated mild cases in prior studies and reports [2].

Further testing and imaging, including CT imaging, revealed that the patient had developed blood clots in the right axillary and brachial veins. Although this occurrence is uncommon in cases of DCS, it is scarcely documented in the literature. Some case reports have mentioned instances of portal and mesenteric vein thromboses being discovered after diving [2,3]. Based on these findings, the patient started anticoagulation therapy with therapeutic heparin. Upon reassessment of the patient's clinical presentation, which did not meet the qualifying criteria for systemic inflammatory response syndrome (SIRS) due to the absence of leukocytosis and fever, alongside a comprehensive imaging review in collaboration with our surgical colleagues, it was concluded that the patient was presented with an acute manifestation of DCS, as evidenced by cutaneous and musculoskeletal symptoms.

Consequently, empiric antibiotic coverage was discontinued. The patient remained on high-flow oxygen therapy with subsequent taper decrement based on continuous clinical and functional assessments, improvement in pain tolerance and symptomatic control, and return of baseline functional status. Remarkably, this treatment strategy effectively mitigated the nitrogenous gas burden within the patient's vascular system without the sequelae commonly associated with DCS, such as neurological impairment, peripheral neuropathy, or cerebrovascular events. The patient was discharged four days after admission, showing a restored range of motion and no signs of neurovascular compromise in the right upper extremity.

## Discussion

DCS arises from the departure of depressurized gas, typically nitrogen, from tissues, forming obstructive bubbles and impeding circulation. This condition is commonly triggered by rapid tissue decompression during swift ascent from deep-sea diving, unpressurized aircraft flight, and extravehicular activities in space [2]. Experts have classified the manifestations of this disorder into three types: type I DCS presents with skin, musculoskeletal, or lymphatic involvement; type II DCS manifests with symptoms of brain or spinal injury; and type III DCS presents with potentially fatal pulmonary complications [2].

Various factors, including dehydration, patent foramen ovale, prior injury, cold ambient temperature, high body fat composition, and recent alcohol consumption, can influence the risk of DCS in individuals. DCS can be subdivided into different variants based on their clinical presentation and systemic effects. Type I DCS presents symptoms related to the skin, lymphatic system, or musculoskeletal system and represents the most prevalent form of this condition. On the other hand, type II DCS affects the nervous system and is linked to the presence of venous bubbles with right-to-left shunting [4,5].

Approximately 75% of individuals with DCS exhibit symptoms within one hour following the triggering event. Healthcare providers must conduct a prompt, targeted assessment and initiate immediate measures for stabilization. Even in cases where oxygen saturation levels are normal, it is advisable to administer high-flow oxygen to suspected DCS patients, as it can aid in the elimination of nitrogen gas pockets in the tissues [2].

The clinical history of DCS typically includes rapid decompression, followed by manifestations in the skin, muscles, bones, joints, inner ear, brain, spine, and, rarely, the lungs. These symptoms may arise within minutes to several hours. Type I DCS commonly affects the shoulder joint, although any joint can be involved. Cutis marmorata, which can be localized or widespread, may also be present. Additionally, patients may exhibit lymph node swelling and pain. Type II DCS may present with the symptoms mentioned above, as well as headache, visual and hearing impairment, nausea, tinnitus, poor coordination, and, in some cases, altered sensorium [6,7]. Our patient's presentation, consisting of severe musculoskeletal and joint pain with swelling and without neurological compromise, aligns with the symptoms of type I DCS. However, the rare adjunctive presentation of coagulopathy in the axillary and brachial veins is an uncommon finding not typically seen in type I DCS [3-5]. Although our patient properly ascended from his dive, his prior injury to the right arm likely was a predisposing factor for his development of DCS. One could hypothesize that a disruption in the soft tissues from the fall on the right shoulder served as a nidus for gas bubbles to escape.

Indeed, DCS in recreational scuba divers is low, approximately 0.4 to one case per 10,000 dives, primarily due to increased awareness and education about prevention in recent years [6]. The most common presenting symptom of DCS is musculoskeletal pain, much like our patient experienced, followed by patchy paresthesia, motor weakness, and constitutional symptoms such as headache and fatigue. Ischemia and inflammation typically cause widespread tissue damage [6]. Cutis marmorata, characterized by pruritic, netlike, red lesions, can develop in the skin. Patients may also experience pain in areas affected by bone and joint necrosis. Additionally, swollen and painful lymph nodes may be observed [7,8]. Symptoms are often vague and non-discrete. However, practitioners should maintain a healthy level of suspicion in patients who present with the above symptoms following a recent dive. Compliance with recommended dive tables, or ascension rates dependent on maximum dive depth, is the most effective way to reduce the risk of DCS, but this does not eliminate the risk [9,10]. This could explain why our patient suffered from DCS despite ascending correctly, according to the recommended guidelines. Other risk factors for DCS include dives deeper than 21 meters, dives conducted in cold water, dives in which divers physically exert themselves more than typical, and a history of DCS [9,10]. For our patient, age, recent illness, including recent hospitalization for nephrolithiasis, and immersion in cold fall-winter waters at depths above 21 meters were proponents for his development of DCS.

The diagnosis of DCS remains clinical mainly, as there are no specific lab values that definitively point towards the diagnosis. Imaging may suggest the diagnosis, but often, these measures do not meaningfully contribute to decisions about treatment and may even delay the diagnosis further [7,8]. Type I DCS may even be confused with systemic viral illness, systemic lupus erythematosus, presentations indicative of acute bacterial diseases such as gonorrhea and syphilis, new presentations of leukemia or lymphoma, and dehydration [11,12]. Type II DCS may be confused on initial clinical presentation to disease processes involving thermal stress, nitrogen narcosis, meningococcemia, multiple sclerosis, hypoglycemia, acute stroke, or inner ear barotrauma [11,12].

Treatment for DCS is often based on the severity of the patient's presentation, but standard measures include oxygen supplementation with the highest possible fraction of inspired oxygen available [2,13]. A definitive treatment for DCS is recompression in a hyperbaric chamber to reduce bubble volume by enhancing the diffusion gradient for inert gas from bubbles to dissolution in blood [2,13,14]. A consensus has emerged that patients with "mild" DCS, primarily defined as musculoskeletal, cutaneous, or constitutional symptoms, can be effectively treated with oxygen supplementations without simultaneous recompression [2,14]. Our patient's clinical course adds evidence to this claim, as the patient presented with symptoms consistent with mild DCS and was effectively treated with 100% oxygen supplementation. However, it is strongly recommended that patients with any signs of focal neurologic deficits undergo recompression therapy as urgently as possible, and this is often complicated by the fact that divers do not present with symptoms until one to two days following their dive [2,14,15]. Additionally, there are contraindications to hyperbaric oxygen therapy, namely, ear injury, pneumothorax, and any condition in which lung collapse may be possible [8]. These conditions may concurrently manifest in divers as DCS, which presents a clinical fork in the road.

Traditional, definitive management for DCS involves immediate administration of 100% oxygen, with subsequent transfer to a hyperbaric oxygen chamber facility if patients show signs of neurologic or respiratory symptoms [14-16]. Our patient exhibited solely musculoskeletal symptoms, so he was properly and adequately managed with 15 L/minute of high-flow oxygen supplementation. Significantly, our patient did not suffer lasting sequelae of DCS or develop new or worsening symptoms.

## Conclusions

Our case highlights the necessity of maintaining a heightened level of clinical suspicion for DCS in individuals who have recently participated in dives or air travel. Compression sickness ensued despite the patient's extensive diving experience, adherence to proper ascending procedures, and preventive measures taken to prevent the condition. This was likely attributed to a predisposing shoulder trauma sustained days before the dive. Although the differential diagnosis was diligently broadened to incorporate the possibility of necrotizing fasciitis, the most probable clinical scenario pointed toward DCS. Owing to a high level of suspicion for this diagnosis, the patient received suitable treatment through high-flow oxygen supplementation, thereby avoiding enduring or long-term sequelae associated with gas embolism.

The treatment protocol for DCS is contingent upon the severity of the patient's condition. The established standard involves the administration of oxygen, aiming for the highest possible fraction of inspired oxygen. Recompression in a hyperbaric oxygen chamber is the definitive therapy for DCS, facilitating the elimination of nitrogenous gas bubbles from the circulatory system and assisting in the dissolution of blood. The current consensus generally agrees that patients with "mild" DCS, characterized by musculoskeletal, cutaneous, or constitutional symptoms, can be effectively managed with oxygen supplementation alone, provided there is no clinical progression or development of new symptoms. However, if such progression or development occurs despite adequate oxygen supplementation, transfer to a facility capable of recompression therapies is warranted. This case underscores three crucial considerations for clinicians managing DCS: (1) It is necessary to consider DCS even when adhering to proper diving protocols; (2) the potential efficacy of high-flow oxygen therapy in the absence of hyperbaric chambers; and (3) the imperative need for a high index of suspicion for DCS in divers presenting with musculoskeletal symptoms in the absence of neurological and infectious causes.
